# *n*-Butyl 2-(2,4-dichloro­anilino)-4,4-dimethyl-6-oxocyclo­hex-1-enecarbo­dithio­ate

**DOI:** 10.1107/S1600536809036320

**Published:** 2009-09-12

**Authors:** El Sayed H. El Ashry, Mohammed R. Amer, Muhammad Raza Shah, Seik Weng Ng

**Affiliations:** a H. E. J. Research Institute of Chemistry, International Center for Chemical and Biological Sciences, University of Karachi, Karachi 75270, Pakistan; b Department of Chemistry, University of Malaya, 50603 Kuala Lumpur, Malaysia

## Abstract

The cyclo­hexene ring in the title compound, C_19_H_23_Cl_2_NOS_2_, adopts an envelope conformation, with the C atom bearing the two methyl groups representing the flap. This atom deviates by 0.630 (2) Å from the plane passing through the other five atoms of the ring (r.m.s. deviation = 0.020 Å). The mol­ecular conformation is stabilized by an intra­molecular N—H⋯S hydrogen bond.

## Related literature

For the crystal structures of the *n*-undeca­nyl and 2-hydroxy­ethyl analogues, see: El Ashry *et al.* (2009*a* ▶,*b* ▶).
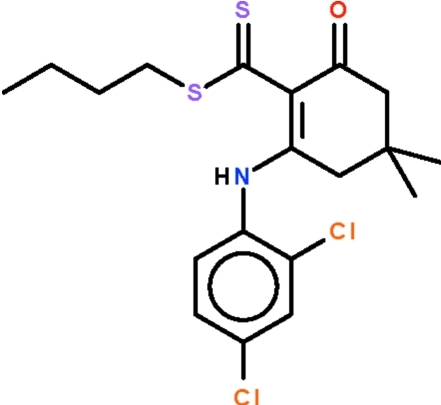


## Experimental

### 

#### Crystal data

C_19_H_23_Cl_2_NOS_2_
*M* *_r_* = 416.40
Monoclinic, 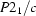

*a* = 9.0321 (1) Å
*b* = 19.4422 (2) Å
*c* = 11.4700 (1) Å
β = 100.331 (1)°
*V* = 1981.52 (3) Å^3^
*Z* = 4
Mo *K*α radiation
μ = 0.55 mm^−1^
*T* = 123 K
0.30 × 0.20 × 0.10 mm

#### Data collection

Bruker SMART APEX diffractometer
Absorption correction: multi-scan (*SADABS*; Sheldrick, 1996 ▶) *T*_min_ = 0.853, *T*_max_ = 0.947
18747 measured reflections
4554 independent reflections
4279 reflections with *I* > 2σ(*I*)
*R*_int_ = 0.016

#### Refinement

*R*[*F* ^2^ > 2σ(*F* ^2^)] = 0.025
*wR*(*F* ^2^) = 0.070
*S* = 1.00
4554 reflections
230 parameters
H atoms treated by a mixture of independent and constrained refinement
Δρ_max_ = 0.41 e Å^−3^
Δρ_min_ = −0.25 e Å^−3^

### 

Data collection: *APEX2* (Bruker, 2008 ▶); cell refinement: *SAINT* (Bruker, 2008 ▶); data reduction: *SAINT*; program(s) used to solve structure: *SHELXS97* (Sheldrick, 2008 ▶); program(s) used to refine structure: *SHELXL97* (Sheldrick, 2008 ▶); molecular graphics: *X-SEED* (Barbour, 2001 ▶); software used to prepare material for publication: *publCIF* (Westrip, 2009 ▶).

## Supplementary Material

Crystal structure: contains datablocks global, I. DOI: 10.1107/S1600536809036320/ci2904sup1.cif

Structure factors: contains datablocks I. DOI: 10.1107/S1600536809036320/ci2904Isup2.hkl

Additional supplementary materials:  crystallographic information; 3D view; checkCIF report

## Figures and Tables

**Table 1:** Hydrogen-bond geometry (Å, °)

*D*—H⋯*A*	*D*—H	H⋯*A*	*D*⋯*A*	*D*—H⋯*A*
N1—H1⋯S2	0.91 (2)	2.08 (2)	2.885 (1)	147 (2)

**Fig. 1.**
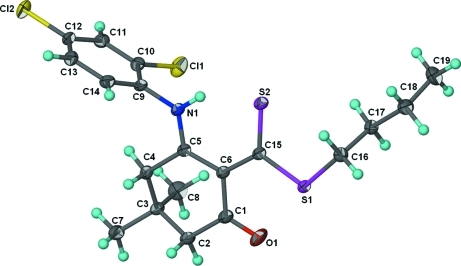
Displacement ellipsoid plot (Barbour, 2001) plot of C19H23Cl2NOS2 at the 70% probability level; H atoms are drawn as spheres of arbitrary radius.

## supplementary crystallographic information

### Experimental

A cooled (283 K) solution of (2,4-dichloroanilino)-4,4-dimethyl-6-oxocyclohex-1-ene (0.2 mol) and sodium hydroxide (0.2 mol) in DMSO (30 ml) and water (2 ml), was treated with carbon disulfide (0.3 mol). After 40 min, *n*-bromobutane (0.15 mol) was added and the reaction mixture was left overnight. The mixture was then diluted with water (200 ml) and acidified with 10% hydrochloric acid. The product was purified on silica gel column chromatography to give yellow crystals when recrystallized from ethanol (m.p. 401 K).

### Refinement

The N-bound H atom was located in a difference Fourier map and was refined freely. C-bound H atoms were placed in calculated positions (C—H = 0.95–0.99 Å) and were included in the refinement in the riding model approximation, with *U_iso_*(H) set to 1.2 to 1.5*U_eq_*(C).

### Crystal data

**Table:**

C_19_H_23_Cl_2_NOS_2_	*F*(000) = 872
*M**_r_* = 416.40	*D*_x_ = 1.396 Mg m^−^^3^
Monoclinic, *P*2_1_/*c*	Mo *K*α radiation, λ = 0.71073 Å
Hall symbol: -P 2ybc	Cell parameters from 9957 reflections
*a* = 9.0321 (1) Å	θ = 2.5–28.2°
*b* = 19.4422 (2) Å	µ = 0.55 mm^−^^1^
*c* = 11.4700 (1) Å	*T* = 123 K
β = 100.331 (1)°	Block, yellow
*V* = 1981.52 (3) Å^3^	0.30 × 0.20 × 0.10 mm
*Z* = 4	

### Data collection

**Table:**

Bruker SMART APEX diffractometer	4554 independent reflections
Radiation source: fine-focus sealed tube	4279 reflections with *I* > 2σ(*I*)
graphite	*R*_int_ = 0.016
ω scans	θ_max_ = 27.5°, θ_min_ = 2.1°
Absorption correction: multi-scan (*SADABS*; Sheldrick, 1996)	*h* = −11→11
*T*_min_ = 0.853, *T*_max_ = 0.947	*k* = −25→25
18747 measured reflections	*l* = −14→14

### Refinement

**Table:**

Refinement on *F*^2^	Primary atom site location: structure-invariant direct methods
Least-squares matrix: full	Secondary atom site location: difference Fourier map
*R*[*F*^2^ > 2σ(*F*^2^)] = 0.025	Hydrogen site location: inferred from neighbouring sites
*wR*(*F*^2^) = 0.070	H atoms treated by a mixture of independent and constrained refinement
*S* = 1.00	*w* = 1/[σ^2^(*F*_o_^2^) + (0.0405*P*)^2^ + 0.9836*P*] where *P* = (*F*_o_^2^ + 2*F*_c_^2^)/3
4554 reflections	(Δ/σ)_max_ = 0.001
230 parameters	Δρ_max_ = 0.41 e Å^−^^3^
0 restraints	Δρ_min_ = −0.25 e Å^−^^3^

### Special details

**Table:**

Geometry. All e.s.d.'s (except the e.s.d. in the dihedral angle between two l.s. planes) are estimated using the full covariance matrix. The cell e.s.d.'s are taken into account individually in the estimation of e.s.d.'s in distances, angles and torsion angles; correlations between e.s.d.'s in cell parameters are only used when they are defined by crystal symmetry. An approximate (isotropic) treatment of cell e.s.d.'s is used for estimating e.s.d.'s involving l.s. planes.

### Fractional atomic coordinates and isotropic or equivalent isotropic displacement parameters (Å^2^)

**Table:**

	*x*	*y*	*z*	*U*_iso_*/*U*_eq_	
Cl1	0.66246 (3)	0.420713 (17)	0.99199 (3)	0.02548 (8)	
Cl2	1.25630 (3)	0.391875 (17)	1.00782 (3)	0.02572 (8)	
S1	0.17044 (3)	0.547076 (14)	0.56447 (2)	0.01521 (7)	
S2	0.45310 (3)	0.595199 (14)	0.71292 (3)	0.01818 (8)	
O1	0.18119 (12)	0.41970 (5)	0.52118 (12)	0.0435 (3)	
N1	0.65717 (11)	0.47990 (5)	0.74995 (8)	0.01551 (19)	
H1	0.625 (2)	0.5237 (10)	0.7584 (16)	0.033 (4)*	
C1	0.31311 (14)	0.40793 (6)	0.56335 (11)	0.0194 (2)	
C2	0.37252 (13)	0.33742 (6)	0.54278 (10)	0.0176 (2)	
H2A	0.4118	0.3381	0.4676	0.021*	
H2B	0.2878	0.3044	0.5331	0.021*	
C3	0.49613 (12)	0.31158 (6)	0.64090 (10)	0.0150 (2)	
C4	0.61567 (12)	0.36787 (6)	0.66192 (10)	0.0163 (2)	
H4A	0.6925	0.3547	0.7312	0.020*	
H4B	0.6662	0.3698	0.5922	0.020*	
C5	0.55899 (12)	0.43880 (6)	0.68327 (9)	0.0133 (2)	
C6	0.41136 (12)	0.46044 (6)	0.63000 (10)	0.0141 (2)	
C7	0.56586 (14)	0.24599 (6)	0.60044 (11)	0.0203 (2)	
H7A	0.6051	0.2554	0.5277	0.030*	
H7B	0.4890	0.2100	0.5851	0.030*	
H7C	0.6482	0.2306	0.6626	0.030*	
C8	0.43521 (15)	0.29707 (7)	0.75460 (11)	0.0246 (3)	
H8A	0.3590	0.2607	0.7397	0.037*	
H8B	0.3898	0.3390	0.7800	0.037*	
H8C	0.5178	0.2822	0.8170	0.037*	
C9	0.80208 (12)	0.45912 (6)	0.81146 (10)	0.0144 (2)	
C10	0.81793 (13)	0.43077 (6)	0.92496 (10)	0.0160 (2)	
C11	0.95775 (13)	0.40992 (6)	0.98561 (10)	0.0171 (2)	
H11	0.9681	0.3902	1.0624	0.021*	
C12	1.08177 (13)	0.41848 (6)	0.93158 (10)	0.0171 (2)	
C13	1.07029 (13)	0.44805 (6)	0.82040 (10)	0.0187 (2)	
H13	1.1572	0.4543	0.7856	0.022*	
C14	0.92928 (13)	0.46846 (6)	0.76063 (10)	0.0169 (2)	
H14	0.9198	0.4889	0.6845	0.020*	
C15	0.35496 (12)	0.53004 (6)	0.63903 (9)	0.0140 (2)	
C16	0.14330 (13)	0.63819 (6)	0.58501 (10)	0.0162 (2)	
H16A	0.2411	0.6618	0.5885	0.019*	
H16B	0.0737	0.6561	0.5150	0.019*	
C17	0.08095 (13)	0.65650 (6)	0.69585 (10)	0.0169 (2)	
H17A	0.1575	0.6462	0.7669	0.020*	
H17B	−0.0089	0.6280	0.6994	0.020*	
C18	0.03800 (14)	0.73269 (6)	0.69634 (11)	0.0226 (2)	
H18A	0.1254	0.7609	0.6846	0.027*	
H18B	−0.0451	0.7418	0.6292	0.027*	
C19	−0.01100 (17)	0.75431 (7)	0.81137 (13)	0.0308 (3)	
H19A	−0.0374	0.8033	0.8074	0.046*	
H19B	0.0716	0.7464	0.8780	0.046*	
H19C	−0.0988	0.7272	0.8227	0.046*	

### Atomic displacement parameters (Å^2^)

**Table:**

	*U*^11^	*U*^22^	*U*^33^	*U*^12^	*U*^13^	*U*^23^
Cl1	0.01664 (14)	0.03539 (18)	0.02576 (15)	−0.00287 (11)	0.00749 (11)	0.00779 (12)
Cl2	0.01535 (14)	0.03229 (17)	0.02687 (16)	0.00715 (11)	−0.00338 (11)	0.00089 (12)
S1	0.01301 (13)	0.01582 (14)	0.01536 (13)	0.00136 (9)	−0.00128 (10)	−0.00127 (9)
S2	0.01510 (14)	0.01493 (14)	0.02250 (15)	−0.00004 (10)	−0.00210 (11)	−0.00381 (10)
O1	0.0189 (5)	0.0256 (5)	0.0756 (8)	0.0054 (4)	−0.0192 (5)	−0.0213 (5)
N1	0.0130 (4)	0.0142 (5)	0.0177 (5)	−0.0003 (3)	−0.0015 (3)	−0.0014 (4)
C1	0.0162 (5)	0.0169 (5)	0.0229 (6)	0.0000 (4)	−0.0023 (4)	−0.0034 (4)
C2	0.0170 (5)	0.0148 (5)	0.0188 (5)	−0.0003 (4)	−0.0028 (4)	−0.0027 (4)
C3	0.0135 (5)	0.0141 (5)	0.0162 (5)	−0.0014 (4)	0.0000 (4)	0.0001 (4)
C4	0.0123 (5)	0.0148 (5)	0.0210 (5)	−0.0003 (4)	0.0012 (4)	−0.0022 (4)
C5	0.0128 (5)	0.0150 (5)	0.0125 (5)	−0.0017 (4)	0.0028 (4)	0.0006 (4)
C6	0.0122 (5)	0.0145 (5)	0.0150 (5)	−0.0011 (4)	0.0008 (4)	−0.0008 (4)
C7	0.0188 (5)	0.0145 (5)	0.0259 (6)	−0.0003 (4)	−0.0010 (4)	−0.0021 (4)
C8	0.0264 (6)	0.0257 (6)	0.0225 (6)	−0.0027 (5)	0.0066 (5)	0.0050 (5)
C9	0.0124 (5)	0.0139 (5)	0.0156 (5)	−0.0009 (4)	−0.0009 (4)	−0.0023 (4)
C10	0.0138 (5)	0.0169 (5)	0.0175 (5)	−0.0024 (4)	0.0035 (4)	−0.0003 (4)
C11	0.0181 (6)	0.0169 (5)	0.0153 (5)	−0.0003 (4)	0.0002 (4)	0.0009 (4)
C12	0.0128 (5)	0.0178 (5)	0.0186 (5)	0.0025 (4)	−0.0028 (4)	−0.0027 (4)
C13	0.0142 (5)	0.0239 (6)	0.0183 (5)	−0.0002 (4)	0.0037 (4)	−0.0027 (4)
C14	0.0169 (5)	0.0197 (5)	0.0137 (5)	−0.0016 (4)	0.0010 (4)	−0.0012 (4)
C15	0.0126 (5)	0.0166 (5)	0.0125 (5)	−0.0006 (4)	0.0013 (4)	0.0003 (4)
C16	0.0176 (5)	0.0144 (5)	0.0158 (5)	0.0018 (4)	0.0007 (4)	0.0019 (4)
C17	0.0156 (5)	0.0163 (5)	0.0184 (5)	0.0011 (4)	0.0020 (4)	−0.0009 (4)
C18	0.0224 (6)	0.0180 (6)	0.0251 (6)	0.0033 (4)	−0.0016 (5)	−0.0030 (4)
C19	0.0309 (7)	0.0264 (7)	0.0340 (7)	0.0087 (5)	0.0030 (6)	−0.0091 (6)

### Geometric parameters (Å, °)

**Table:**

Cl1—C10	1.7287 (11)	C8—H8A	0.98
Cl2—C12	1.7387 (12)	C8—H8B	0.98
S1—C15	1.7628 (11)	C8—H8C	0.98
S1—C16	1.8094 (12)	C9—C14	1.3903 (16)
S2—C15	1.6851 (11)	C9—C10	1.3973 (16)
O1—C1	1.2244 (16)	C10—C11	1.3885 (16)
N1—C5	1.3291 (14)	C11—C12	1.3840 (17)
N1—C9	1.4293 (14)	C11—H11	0.95
N1—H1	0.911 (18)	C12—C13	1.3856 (17)
C1—C6	1.4733 (15)	C13—C14	1.3916 (16)
C1—C2	1.5061 (16)	C13—H13	0.95
C2—C3	1.5214 (15)	C14—H14	0.95
C2—H2A	0.99	C16—C17	1.5222 (16)
C2—H2B	0.99	C16—H16A	0.99
C3—C4	1.5258 (15)	C16—H16B	0.99
C3—C8	1.5300 (16)	C17—C18	1.5315 (16)
C3—C7	1.5307 (16)	C17—H17A	0.99
C4—C5	1.5063 (15)	C17—H17B	0.99
C4—H4A	0.99	C18—C19	1.5240 (19)
C4—H4B	0.99	C18—H18A	0.99
C5—C6	1.4269 (15)	C18—H18B	0.99
C6—C15	1.4562 (15)	C19—H19A	0.98
C7—H7A	0.98	C19—H19B	0.98
C7—H7B	0.98	C19—H19C	0.98
C7—H7C	0.98		
			
C15—S1—C16	105.02 (5)	C14—C9—N1	120.57 (10)
C5—N1—C9	124.86 (10)	C10—C9—N1	120.21 (10)
C5—N1—H1	115.5 (11)	C11—C10—C9	120.93 (10)
C9—N1—H1	119.6 (11)	C11—C10—Cl1	118.85 (9)
O1—C1—C6	121.90 (11)	C9—C10—Cl1	120.22 (9)
O1—C1—C2	117.19 (11)	C12—C11—C10	118.49 (10)
C6—C1—C2	120.90 (10)	C12—C11—H11	120.8
C1—C2—C3	114.80 (9)	C10—C11—H11	120.8
C1—C2—H2A	108.6	C11—C12—C13	121.94 (11)
C3—C2—H2A	108.6	C11—C12—Cl2	118.20 (9)
C1—C2—H2B	108.6	C13—C12—Cl2	119.85 (9)
C3—C2—H2B	108.6	C12—C13—C14	118.85 (11)
H2A—C2—H2B	107.5	C12—C13—H13	120.6
C2—C3—C4	106.54 (9)	C14—C13—H13	120.6
C2—C3—C8	111.29 (10)	C9—C14—C13	120.55 (11)
C4—C3—C8	110.49 (10)	C9—C14—H14	119.7
C2—C3—C7	109.76 (9)	C13—C14—H14	119.7
C4—C3—C7	109.11 (9)	C6—C15—S2	125.12 (8)
C8—C3—C7	109.58 (10)	C6—C15—S1	116.90 (8)
C5—C4—C3	115.52 (9)	S2—C15—S1	117.97 (6)
C5—C4—H4A	108.4	C17—C16—S1	114.63 (8)
C3—C4—H4A	108.4	C17—C16—H16A	108.6
C5—C4—H4B	108.4	S1—C16—H16A	108.6
C3—C4—H4B	108.4	C17—C16—H16B	108.6
H4A—C4—H4B	107.5	S1—C16—H16B	108.6
N1—C5—C6	123.03 (10)	H16A—C16—H16B	107.6
N1—C5—C4	115.65 (10)	C16—C17—C18	111.18 (10)
C6—C5—C4	121.26 (10)	C16—C17—H17A	109.4
C5—C6—C15	123.74 (10)	C18—C17—H17A	109.4
C5—C6—C1	116.59 (10)	C16—C17—H17B	109.4
C15—C6—C1	119.67 (10)	C18—C17—H17B	109.4
C3—C7—H7A	109.5	H17A—C17—H17B	108.0
C3—C7—H7B	109.5	C19—C18—C17	112.52 (11)
H7A—C7—H7B	109.5	C19—C18—H18A	109.1
C3—C7—H7C	109.5	C17—C18—H18A	109.1
H7A—C7—H7C	109.5	C19—C18—H18B	109.1
H7B—C7—H7C	109.5	C17—C18—H18B	109.1
C3—C8—H8A	109.5	H18A—C18—H18B	107.8
C3—C8—H8B	109.5	C18—C19—H19A	109.5
H8A—C8—H8B	109.5	C18—C19—H19B	109.5
C3—C8—H8C	109.5	H19A—C19—H19B	109.5
H8A—C8—H8C	109.5	C18—C19—H19C	109.5
H8B—C8—H8C	109.5	H19A—C19—H19C	109.5
C14—C9—C10	119.20 (10)	H19B—C19—H19C	109.5
			
O1—C1—C2—C3	−149.43 (13)	C14—C9—C10—C11	−2.07 (17)
C6—C1—C2—C3	31.47 (16)	N1—C9—C10—C11	179.58 (10)
C1—C2—C3—C4	−52.05 (13)	C14—C9—C10—Cl1	178.25 (9)
C1—C2—C3—C8	68.46 (13)	N1—C9—C10—Cl1	−0.10 (15)
C1—C2—C3—C7	−170.06 (10)	C9—C10—C11—C12	0.65 (17)
C2—C3—C4—C5	52.18 (12)	Cl1—C10—C11—C12	−179.66 (9)
C8—C3—C4—C5	−68.83 (13)	C10—C11—C12—C13	1.04 (17)
C7—C3—C4—C5	170.62 (9)	C10—C11—C12—Cl2	−179.71 (9)
C9—N1—C5—C6	175.52 (10)	C11—C12—C13—C14	−1.26 (18)
C9—N1—C5—C4	−7.29 (16)	Cl2—C12—C13—C14	179.50 (9)
C3—C4—C5—N1	151.63 (10)	C10—C9—C14—C13	1.84 (17)
C3—C4—C5—C6	−31.13 (15)	N1—C9—C14—C13	−179.82 (10)
N1—C5—C6—C15	2.50 (17)	C12—C13—C14—C9	−0.21 (17)
C4—C5—C6—C15	−174.53 (10)	C5—C6—C15—S2	−0.17 (16)
N1—C5—C6—C1	−177.12 (10)	C1—C6—C15—S2	179.44 (9)
C4—C5—C6—C1	5.84 (15)	C5—C6—C15—S1	178.79 (8)
O1—C1—C6—C5	174.85 (13)	C1—C6—C15—S1	−1.60 (14)
C2—C1—C6—C5	−6.09 (16)	C16—S1—C15—C6	−175.86 (8)
O1—C1—C6—C15	−4.79 (19)	C16—S1—C15—S2	3.18 (8)
C2—C1—C6—C15	174.27 (10)	C15—S1—C16—C17	−89.34 (9)
C5—N1—C9—C14	95.10 (14)	S1—C16—C17—C18	−170.96 (8)
C5—N1—C9—C10	−86.58 (14)	C16—C17—C18—C19	−174.62 (10)

### Hydrogen-bond geometry (Å, °)

**Table:**

*D*—H···*A*	*D*—H	H···*A*	*D*···*A*	*D*—H···*A*
N1—H1···S2	0.91 (2)	2.08 (2)	2.885 (1)	147 (2)
